# Maternal and fetal genetic predispositions to insulin deficiency and resistance affect fetal growth through distinct pathways

**DOI:** 10.1007/s00125-026-06669-7

**Published:** 2026-02-03

**Authors:** Gechang Yu, Claudia H. T. Tam, Mai Shi, Alice E. Hughes, Chuiguo Huang, Yuzhi Deng, Michael N. Weedon, Cadmon K. P. Lim, Chi Chiu Wang, Juliana C. N. Chan, Wing Hung Tam, William Lowe, Rachel M. Freathy, Richard A. Oram, Ronald C. W. Ma

**Affiliations:** 1 Department of Medicine and Therapeutics, The Chinese University of Hong Kong, Hong Kong, China; 2 Laboratory for Molecular Epidemiology in Diabetes, Li Ka Shing Institute of Health Sciences, The Chinese University of Hong Kong, Hong Kong, China; 3 Chinese University of Hong Kong-Shanghai Jiao Tong University Joint Research Centre in Diabetes Genomics and Precision Medicine, Hong Kong Institute of Diabetes and Obesity, The Chinese University of Hong Kong, Hong Kong, China; 4 Department of Clinical and Biomedical Sciences, Faculty of Health and Life Sciences, University of Exeter Medical School, University of Exeter, Exeter, UK; 5 Department of Obstetrics and Gynaecology, The Chinese University of Hong Kong, Hong Kong, China; 6 Li Ka Shing Institute of Health Sciences, The Chinese University of Hong Kong, Hong Kong, China; 7 CUHK Medical Centre, Shatin, New Territories Hong Kong, China; 8 Department of Medicine, Feinberg School of Medicine, Northwestern University, Chicago, IL USA

**Keywords:** Fetal growth, Insulin deficiency, Insulin resistance, Mendelian randomisation, Multi-ethnic populations, Partitioned polygenic risk scores, Type 2 diabetes

## Abstract

**Aims/hypothesis:**

We aimed to investigate whether maternal and fetal genetic predispositions to insulin deficiency and resistance affect offspring fetal growth through distinct pathways in multi-ethnic populations.

**Methods:**

In 5065 multi-ethnic mother–infant pairs, we examined the conditional associations of maternal and fetal partitioned polygenic risk scores (pPRSs) for type 2 diabetes-related pathways with fetal growth outcomes, including birthweight, sum of skinfold thicknesses (SSF), large-for-gestational-age (LGA) births and small-for-gestational-age (SGA) births. Two-sample Mendelian randomisation (2SMR) in Europeans was performed for triangulation. Exposures were eight type 2 diabetes-related pathways (*n*=1,812,017), eight beta cell function indices (*n*=26,356) and two insulin sensitivity indices (*n*=53,657). Outcomes were maternal and fetal genetically determined birthweight (*n*=406,063). Mediation analysis was used to assess the mediation effects of maternal glucose levels and BMI on maternal genetic effects and of cord blood C-peptide on fetal genetic effects. Co-localisation analyses were performed to test for shared causal variants.

**Results:**

Fetal type 2 diabetes polygenic risk score (PRS) and pPRSs for lipodystrophy-related insulin resistance and impaired fasting glucose (IFG)-related insulin deficiency were associated with lower birthweight and SSF, while maternal type 2 diabetes PRS and pPRSs for IFG-related insulin deficiency and obesity-related insulin resistance were associated with higher offspring birthweight, SSF and LGA. These associations were consistent across five ethnic groups. Maternal post-load hyperglycaemia mediated 44.2% and 34.2% of the effects of type 2 diabetes PRS and IFG pPRS, respectively, while maternal BMI mediated 43.4% of the effect of Obesity pPRS. 2SMR found consistent results in Europeans and further revealed that fetal insulin sensitivity index and corrected insulin response were associated with higher birthweight. Some loci with shared causal variants acted through multiple pathways, including *CDKAL1*, *TCF7L2*, *ADCY5* and *MACF1*.

**Conclusions/interpretation:**

Reduced fetal growth may be driven by lipodystrophy-related insulin resistance and IFG-related insulin deficiency pathways. Targeting pregnant women with high type 2 diabetes PRS/pPRS and prescribing interventions to reduce their post-load hyperglycaemia and BMI may help reduce offspring risk of LGA.

**Graphical Abstract:**

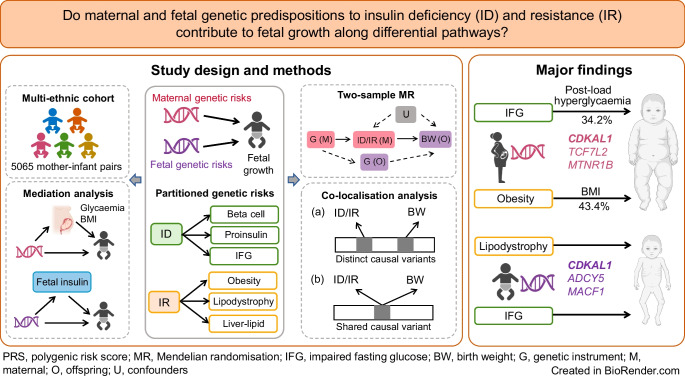

**Supplementary Information:**

The online version contains peer-reviewed but unedited supplementary material available at 10.1007/s00125-026-06669-7.


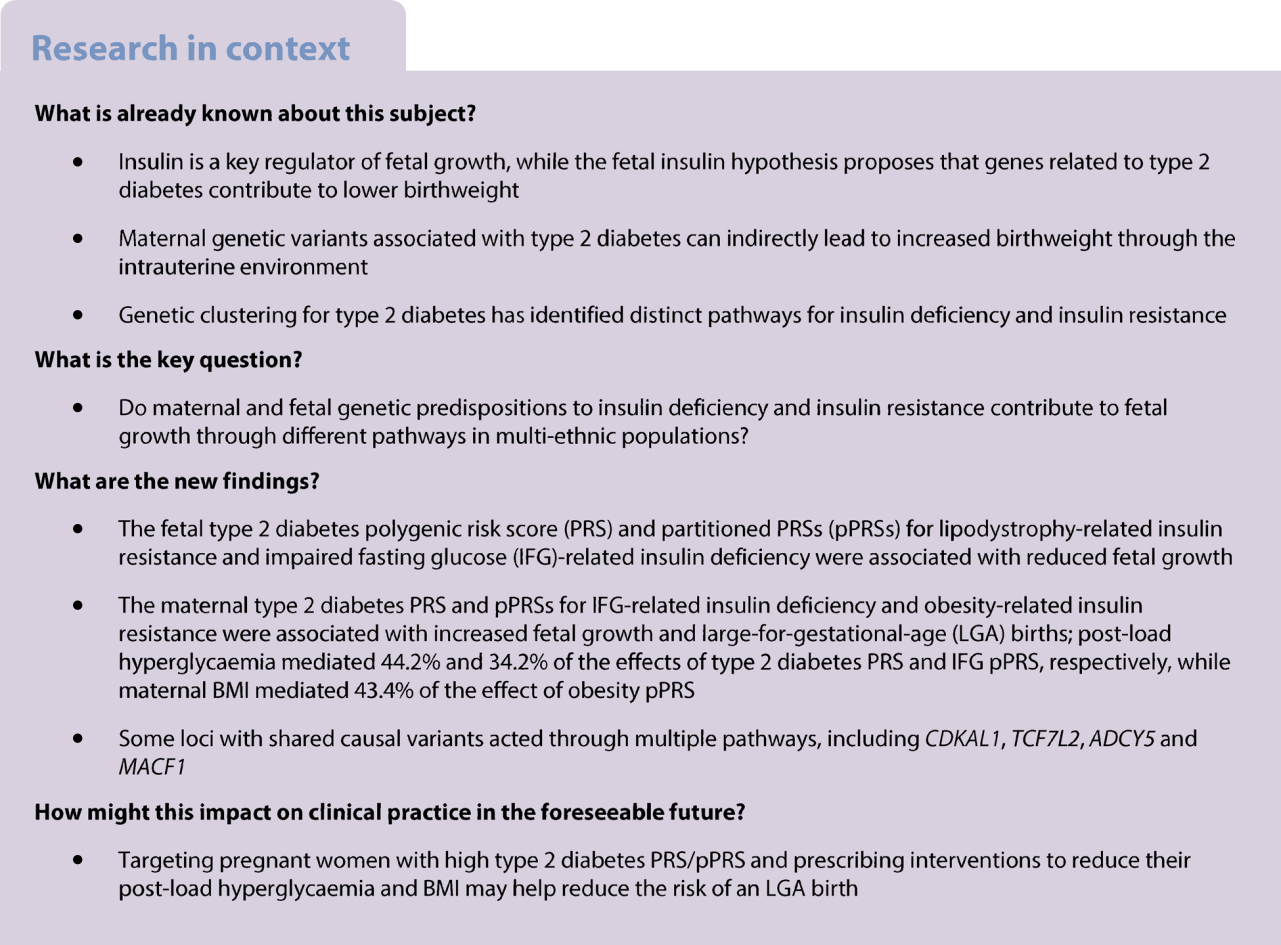


## Introduction

Maternal and fetal genotypes both contribute to fetal growth [1–6]. Fetal insulin is a key regulator of fetal growth [7, 8]. The fetal insulin hypothesis [9, 10] proposed that fetal genes related to insulin deficiency and insulin resistance result in reduced insulin-mediated fetal growth and an increased risk of type 2 diabetes in later life [8]. Recent studies have found associations between fetal insulin-deficiency genetic variants and lower birthweight in European populations [4, 8, 11]. In addition, maternal genetic variants associated with type 2 diabetes can indirectly lead to increased birthweight through elevated maternal glucose (intrauterine environment) [1, 2]. However, little is known about whether both maternal and fetal insulin deficiency/insulin resistance genetic variants can affect fetal growth in non-European populations; this is important for understanding the genetic mechanisms of insulin-mediated fetal growth in under-represented populations.

Furthermore, previous studies investigating the associations between type 2 diabetes and glucose genetic variants and birthweight [1, 2, 12, 13] have not considered that type 2 diabetes as well as insulin deficiency and insulin resistance are multifactorial and heterogeneous phenotypes [14–17] that can be attributed to multiple biological pathways. Recently developed multi-ancestry genetic clustering and partitioned polygenic risk scores (pPRSs) for type 2 diabetes [15, 18] allow us to characterise individual genetic predispositions to different biological pathways of insulin deficiency and insulin resistance in diverse populations [16, 19, 20]. Therefore, differential insulin deficiency and insulin resistance pathways may be implicated in maternal and fetal genetic effects on offspring growth.

The current study aims to investigate the pathways whereby maternal and fetal genetic predispositions to insulin deficiency and insulin resistance affect offspring fetal growth by integrating a multi-ethnic cohort study of 5065 mother–infant pairs with two-sample Mendelian randomisation (2SMR) analyses in Europeans. In addition, we assessed whether maternal glucose levels and BMI mediated maternal genetic effects, and whether fetal insulin mediated fetal genetic effects. Co-localisation analysis was performed to pinpoint the shared causal variant between insulin deficiency/resistance and birthweight.

## Methods

### Study participants

The Hyperglycaemia and Adverse Pregnancy Outcome (HAPO) Study is a multicentre, international study, which recruited over 25,000 pregnant women across 15 centres in nine countries and collected phenotypes related to maternal glucose metabolism and fetal growth. This current study utilised available data from 6636 mother–infant pairs of five ethnic groups from the original HAPO Study, including 1507 European pairs from four field centres (Belfast, Brisbane, Newcastle and Toronto), 1345 African Caribbean pairs from Barbados, 889 Mexican American pairs from California, 1253 Thai pairs from Bangkok and 1642 Chinese pairs from Hong Kong. Details of the HAPO Study have been described previously [21]. The HAPO Study protocol was approved by the Institutional Review Board of each field centre, and written informed consent was given by each mother. The HAPO-HK study received ethical approval from the Clinical Research Ethics Committee of the Chinese University of Hong Kong (CRE-2002.119, CRE-2008.017, CRE-2013.042, CRE2015.473). Clinical characteristics of mothers during the OGTT and offspring at birth in each ethnic group are described in Table 1.

**Table 1:** Characteristics of HAPO Study mothers (during OGTT) and offspring (at birth) in each ethnic group

Characteristic	European	Mexican American	African Caribbean	Chinese	Thai
N	1507	889	1345	1642	1253
Maternal age at OGTT, years	31.3 ± 5.3	29.1 ± 5.4	25.6 ± 5.7	30.7 ± 4.9	27.8 ± 5.5
Gestational age at OGTT, weeks	28.5 ± 1.4	26.9 ± 2.1	27 ± 1.8	27.7 ± 1.3	28.2 ± 1.8
Height at OGTT, cm	164.2 ± 6.4	159.7 ± 5.8	163.9 ± 6.7	158.4 ± 5.2	153.7 ± 5.5
Weight at OGTT, kg	76.8 ± 14	76.9 ± 15.6	74.7 ± 16.6	61.4 ± 8.3	60.7 ± 9.2
BMI at OGTT, kg/m^2^	28.5 ± 4.8	30.1 ± 5.6	27.8 ± 6	24.5 ± 3	25.7 ± 3.6
Weight before pregnancy, kg	66.2 ± 14.2	68.9 ± 15.5	65.6 ± 15.4	52 ± 7.8	51.8 ± 8.8
BMI before pregnancy, kg/m^2^	24.5 ± 5	27 ± 5.6	24.4 ± 5.6	20.7 ± 2.9	21.9 ± 3.5
Systolic BP, mmHg	108.5 ± 9.9	108.4 ± 10.2	103.3 ± 10	101.1 ± 9.8	104.1 ± 9.9
Diastolic BP, mmHg	71.3 ± 8	71.8 ± 8.1	67.2 ± 8	63.4 ± 7.3	67.6 ± 7.7
FPG, mmol/l	4.6 ± 0.4	4.7 ± 0.4	4.5 ± 0.4	4.4 ± 0.4	4.4 ± 0.4
1 h glucose, mmol/l	7.3 ± 1.6	7.7 ± 1.9	6.8 ± 1.5	7.7 ± 1.7	8.2 ± 1.7
2 h glucose, mmol/	6.1 ± 1.2	6.2 ± 1.4	6.1 ± 1.2	6.6 ± 1.3	6.6 ± 1.4
HbA_1c_, mmol/l	28.6 ± 4.2	29.3 ± 3.9	28.8 ± 5.1	30.5 ± 4.0	24.7 ± 6.9
HbA_1c_, %	4.8 ± 0.4	4.8 ± 0.4	4.8 ± 0.5	4.9 ± 0.4	4.4 ± 0.6
FCP, μg/l	2 ± 0.8	2.4 ± 1	1.9 ± 1.2	1.8 ± 0.6	1.6 ± 0.8
Gestational diabetes, n (%)	224 (14.9%)	230 (25.9%)	140 (10.4%)	236 (14.4%)	273 (21.8%)
Previous parity ≥1, n (%)	639 (42.4%)	659 (74.1%)	643 (39.2%)	573 (45.7%)	649 (48.3%)
Gestational age at delivery, weeks	39.9 ± 1.2	39.7 ± 1.2	39.7 ± 1.2	39.3 ± 1.5	39.4 ± 1.3
Birth sex male, n (%)	753 (50%)	452 (50.8%)	695 (51.7%)	858 (52.3%)	619 (49.4%)
Birthweight, g	3422.7 ± 503.9	3432.2 ± 427.1	3224.8 ± 453.3	3164.9 ± 428.3	3103.1 ± 391.9
Birth length, cm	50.6 ± 2.2	50.6 ± 1.7	49.3 ± 2.5	49.1 ± 1.9	49.5 ± 1.5
SSF, mm	12.9 ± 2.7	14.2 ± 2.9	11.5 ± 1.8	11.8 ± 2.3	11.7 ± 2.4
Cord blood C-peptide, μg/l	1 ± 0.6	1.1 ± 0.6	1 ± 0.5	1 ± 0.6	1 ± 0.6

Data are expressed as mean ± SD or *n* (%)

The proportion of previous parity ≥1 is calculated in mothers with self-reported previous parity information

### Clinical and laboratory measurements

All women underwent a 75 g OGTT at gestational age 24–32 weeks (close to 28 weeks). Maternal fasting plasma glucose (FPG), 1 h glucose (GLU60) and 2 h glucose (GLU120), fasting C-peptide (FCP) and HbA_1c_ were measured during maternal OGTT. Pre-pregnancy weight was self-reported and collected by the questionnaire. Maternal DNA was collected during the OGTT visit, except for the Hong Kong samples, which were obtained at the follow-up visit at approximately 7 years postpartum [22]. Fetal DNA and C-peptide were collected from the umbilical cord within 5 min of delivery. The AUC for glucose (GAUC) during OGTT at 0–120 min was calculated using the trapezoid rule. HOMA2-B and HOMA2-IR were calculated by the web-based HOMA2 calculator based on the updated HOMA2 model [23] to evaluate beta cell function (BCF) and insulin resistance. Offspring fetal growth, including birth length, birthweight, head circumference (HCC) and skinfold thickness (flank, triceps and subscapular), was measured within 72 h of birth using methods and equipment standardised across all field centres [24]. Sum of skinfold thicknesses (SSF) was summed by the flank, triceps and subscapular skinfolds to assess neonatal fat levels. Ponderal index (PI), a proxy measure of adiposity and fetal growth restraint for infants [25, 26], was calculated by weight (kg) divided by cubed height (m^3^) [27]. All study measurements were standardised as part of the HAPO Study protocol.

### Clinical outcomes

The primary outcomes for fetal growth in the HAPO Study were birthweight and SSF. We calculated the corrected birthweight [28] adjusted for gestational age and sex within each HAPO ethnic group (including participants without genetic data). European infant data were additionally adjusted for field centre. The secondary outcomes included small-for-gestational-age (SGA) birth defined as corrected birthweight <10th centile in each group, and large-for-gestational-age (LGA) birth defined as corrected birthweight >90th centile in each group. For the numbers of case and control and corrected birthweight thresholds for SGA and LGA births in each HAPO group, please see electronic supplementary material (ESM) Table 1.

### Genotyping, quality control and imputation

Maternal and fetal DNA samples were genotyped using the following arrays: (1) HumanOmniZhongHua-8 BeadChip (Hong Kong Chinese participants); (2) Omni1-Quad_v1-0_B (Thai participants); (3) Illumina Human610 Quad (European participants); and (4) Human1M Duo (African Caribbean and Mexican American participants). Details of genotyping have been described previously [22, 29]. Genotype data from HAPO centres other than Hong Kong were obtained from the database of Genotypes and Phenotypes (dbGaP), a publicly accessible repository for genetic and phenotypic data. The specific dataset used in this study was entitled ‘Hyperglycaemia and Adverse Pregnancy Outcome (HAPO) Study: maternal glycaemia and birthweight GEI Study’, with accession number phs000096.v4.p1, made available by the HAPO Study steering committee. The dbGaP data downloading followed the guidelines and regulations for data access and usage as outlined and approved by the repository. We applied uniform quality control (QC) procedures on the maternal and fetal genotype data of each ethnic group (for details, see ESM Methods).

Before imputation, we performed genetic ancestry inference on all individuals in each ethnic group following the recommended pipeline from Peterson et al [30] (for details, see ESM Methods and ESM Figs 1, 2). Of note, most Chinese (98.0% of mothers, 99.7% of children) and Thai participants (91.0% of mothers, 98.3% of children) were assigned to East Asian ancestry. QCed maternal and fetal genotype data were imputed to the 1000 Genomes Project phase III reference panel using the Michigan Imputation Server [31]. SNPs with an imputation quality score Rsq <0.5 were excluded in the following analysis. Finally, 5586 mothers and 5310 infants (5065 pairs) with QCed imputed genotype data were included in the analysis. The clinical characteristics of HAPO mothers and infants with genotype data in each ethnic group are listed in ESM Table 2. We did not find differences in clinical characteristics between participants with genotype data and full participants in each group (ESM Table 2).

### pPRS calculation in HAPO Study

In the largest multi-ancestry type 2 diabetes genome-wide association study (GWAS; *n*>2.5 million, 60.3% European ancestry, 19.83% East Asian ancestry, 10.48% African American ancestry, 5.86% Hispanic ancestry, 3.34% South Asian ancestry, 0.19% South African ancestry), conducted by Suzuki et al [15], 1289 independent significant (multi-ancestry) SNPs associated with type 2 diabetes were divided into eight clusters, according to their associations with cardiometabolic traits. For each type 2 diabetes-related pathway, the pPRS in each HAPO group was calculated as the sum of risk alleles, weighted by their effect size for type 2 diabetes using the same set of SNPs. For a more detailed description of each genetic cluster, please refer to ESM Table 3. However, we believe the names of some of those clusters are somewhat confusing or unclear and would benefit from clarification. The ‘residual glycaemic’ cluster is characterised by increased fasting glucose and HbA_1c_, and this cluster was renamed as ‘impaired fasting glucose (IFG)’ in our study for clarity. The ‘metabolic syndrome’ cluster is quite similar to the ‘lipodystrophy’ cluster in showing association with ectopic fat deposition. However, the former is more strongly associated with insulin resistance while the latter is more strongly associated with reduced body and trunk fat. So, we renamed the ‘metabolic syndrome’ cluster as ‘insulin resistance–lipodystrophy’ (IR-lipodystrophy) for clarity.

For the missing SNPs in each HAPO population, we used the LDproxy in LDlink [19] to find proxy SNPs (linkage disequilibrium [LD] *R*^2^>0.8) in the corresponding populations (e.g. African reference panel for African Caribbean data) from the 1000 Genome Project reference panel. A small proportion of participants was not assigned to the predominant genetic ancestry in each ethnic group because they did not reach the predefined threshold of ancestry assignment or were genetically admixed. Since we calculated the multi-ancestry PRSs for each participant, we included all participants in each HAPO ethnic group and performed separate analyses in each ethnic group rather than excluding the participants based on genetic ancestry, to account for ethnicity-specific socioeconomic, cultural and other environmental factors, and to ensure fair clinical application of polygenic risk score (PRS) in genetically admixed participants [32]. We used the *z* score of transformed pPRS (per SD) in each HAPO group [16].

### 2SMR analysis

For triangulation, we performed cluster-stratified 2SMR [33] in European individuals to test the causal effects of maternal and fetal partitioned type 2 diabetes risk for each pathway (exposure) on offspring birthweight (outcome) using the inverse variance-weighted (IVW) method by the R package TwoSampleMR v0.6.9 [34]. Outcomes are unbiased estimates [35] of maternal and fetal genetic effects on offspring birthweight by weighted linear model from a European GWAS meta-analysis [2] using the EGG consortium and UK Biobank data (*n*>400,000). Exposures were type 2 diabetes-related pathways [15] consistent with multi-ancestry pPRSs above. To avoid population stratification, among the 1289 multi-ancestry type 2 diabetes-associated SNPs, only those that were genome-wide significant (*p*<5 × 10^−8^) and independent (*r*^2^<0.05) in the European-specific GWAS (>240,000 cases and >1.5 million controls) from Suzuki et al [15] were selected as genetic instruments for each type 2 diabetes-related pathway. For the missing instrumental variables, we used the LDproxy in LDlink [19] to find proxy SNPs (LD *R*^2^>0.8) in the European populations from the 1000 Genome Project reference panel. Finally, the exposures of type 2 diabetes-related pathways included Beta cell + PI (*n*=79 SNPs), Beta cell − PI (*n*=66), IFG (*n*=209), Obesity (*n*=181), Body fat (*n*=155), Lipodystrophy (*n*=40), IR-lipodystrophy (*n*=125) and Liver/lipid metabolism (*n*=3). The minimum *F* statistic of these instruments was 29. Please refer to ESM Table 4 for detailed information about each instrument.

To better interrogate the causal effects of insulin secretion and sensitivity on birthweight, we additionally performed 2SMR using eight BCF indices from a GWAS of 26,356 European non-diabetic individuals conducted by Madsen et al [36] and two insulin sensitivity indices from a GWAS of 53,657 European non-diabetic individuals conducted by Williamson et al [37] as exposures. The BCF indices included one measured in the fasting state (HOMA-B, SNPs *n*=5), two indicators of disposition (disposition index [DI] [*n*=26] [38] and disposition index for BIGTT-AIR and BIGTT-SI [DIBIG] [*n*=12] [39]) for the ability of the body to dispose of a glucose load, two insulinogenic indices (insulinogenic index [xinsdG30] [*n*=26] [40] and modified insulinogenic index [xinsG30] [*n*=29] [41]) for the early-phase insulin secretory capacity of beta cells and three indicators for the overall insulin response of beta cells (BCF insulin sensitivity GTT for acute insulin response [BIGTT-AIR] [*n*=20] [42], corrected insulin response [CIR] [*n*=25] [39] and Stumvoll [*n*=22] [39]). The insulin sensitivity indices included modified Stumvoll insulin sensitivity index (ISI; *n*=8) [39, 43] and the fold change in insulin concentration (insulin fold change [IFC], *n*=1) following an OGTT. The ISI assessed the whole-body insulin sensitivity, while IFC was indicative of post-challenge insulin resistance. The minimum *F* statistic of these instruments was 27. Please refer to ESM Tables 5, 6 for the formula, loci and instrumental variables of these indices.

To account for horizontal pleiotropy, we employed four additional Mendelian randomisation (MR) methods: (1) median-weighted [44]; (2) Egger [45]; (3) weighted mode [46]; and (4) MR-Pleiotropy Residual Sum and Outlier (PRESSO) [47]. We also tested the heterogeneity using Cochran’s *Q* test (MR-Egger and MR-IVW) and horizontal pleiotropy using MR-Egger intercepts and MR-PRESSO global test.

Furthermore, to assess whether there was a shared causal variant between fetal secretion/resistance and birthweight, we performed co-localisation analysis using the ‘coloc’ R package v6.0.1 [48]. We selected all SNPs 500 kb up- and downstream of each signal to calculate the posterior probability that insulin resistance/secretion and birthweight share the same associated variant (posterior probability of hypothesis 4 [PPH_4_]). PPH_4_ >0.75 was considered to have strong evidence of co-localisation.

### Statistical analysis

For each pathway, to account for the correlation between maternal and fetal genotypes and potential confounding effects, we examined the conditional associations of maternal and fetal pPRS with fetal growth measurements and cord blood C-peptide in each HAPO group. The equation is listed below:

$${Outcome}_{j}= {\beta }_{Mi}\times {PRS}_{Mi}+ {\beta }_{Fi}\times {PRS}_{Fi}+Covariates$$

${\beta }_{Mi}$ quantifies the indirect genetic effect of type 2 diabetes-related pathway *i* from maternal genotypes on fetal growth outcome *j*, while ${\beta }_{Fi}$ quantifies the direct genetic effect of type 2 diabetes-related pathway *i* from fetal genotypes on fetal growth outcome *j*. Covariates included gestational age at delivery, parity, maternal age at delivery, newborn sex and fetal top-five principal components (PCs). European mother–infant pairs were additionally adjusted for field centre. We used the random-effects IVW method in R meta package to meta-analyse the results in all ethnic groups. Power analysis was performed by R metapower package to confirm where there was enough power to detect heterogeneity. The heterogeneity across ethnic groups was assessed by Cochran’s *Q* statistic [49, 50] and Higgins and Thompson’s *I*^2^ statistic [51]. As a rule of thumb [52], *I*^2^>50% and Cochran’s *Q p*<0.05 suggested considerable heterogeneity across ethnic groups in our study.

We next examined the associations of maternal PRS/pPRS with maternal BMI and glucose levels in the third trimester, adjusting for gestational age at OGTT, parity, maternal age, field centre and maternal top-five PCs, and performed mediation analysis to test the mediator role of maternal glucose and BMI on maternal genetic effects on fetal growth by Mediation toolbox [53] in MATLAB with 10,000 bootstrapping. We also performed the mediation analyses to test whether cord blood C-peptide mediated the fetal genetic effects on birthweight. The Bonferroni method was used to correct for multiple comparisons. The statistical significance threshold was *p*<0.0056 (0.05/9) for eight pPRSs and type 2 diabetes PRS. For insulin deficiency and insulin resistance measurements (*n*=10) derived in non-diabetic individuals used in 2SMR analysis, the significance threshold was *p*<0.005.

### Sensitivity analyses

We used another set of newly developed multi-ancestry type 2 diabetes pPRSs (using soft-clustering) from Smith et al [18] to repeat the main analyses as validation. Please see ESM Table 3 for detailed description of the soft-clustering clusters. We performed separate analyses for maternal and fetal PRSs by not adjusting maternal and fetal PRSs simultaneously in the regression models. We also evaluated the interaction effects of maternal glucose levels/BMI and PRS on offspring birthweight in the mediation models.

## Results

### Differential associations of maternal and fetal type 2 diabetes pPRSs with fetal growth

**Fig. 1:**
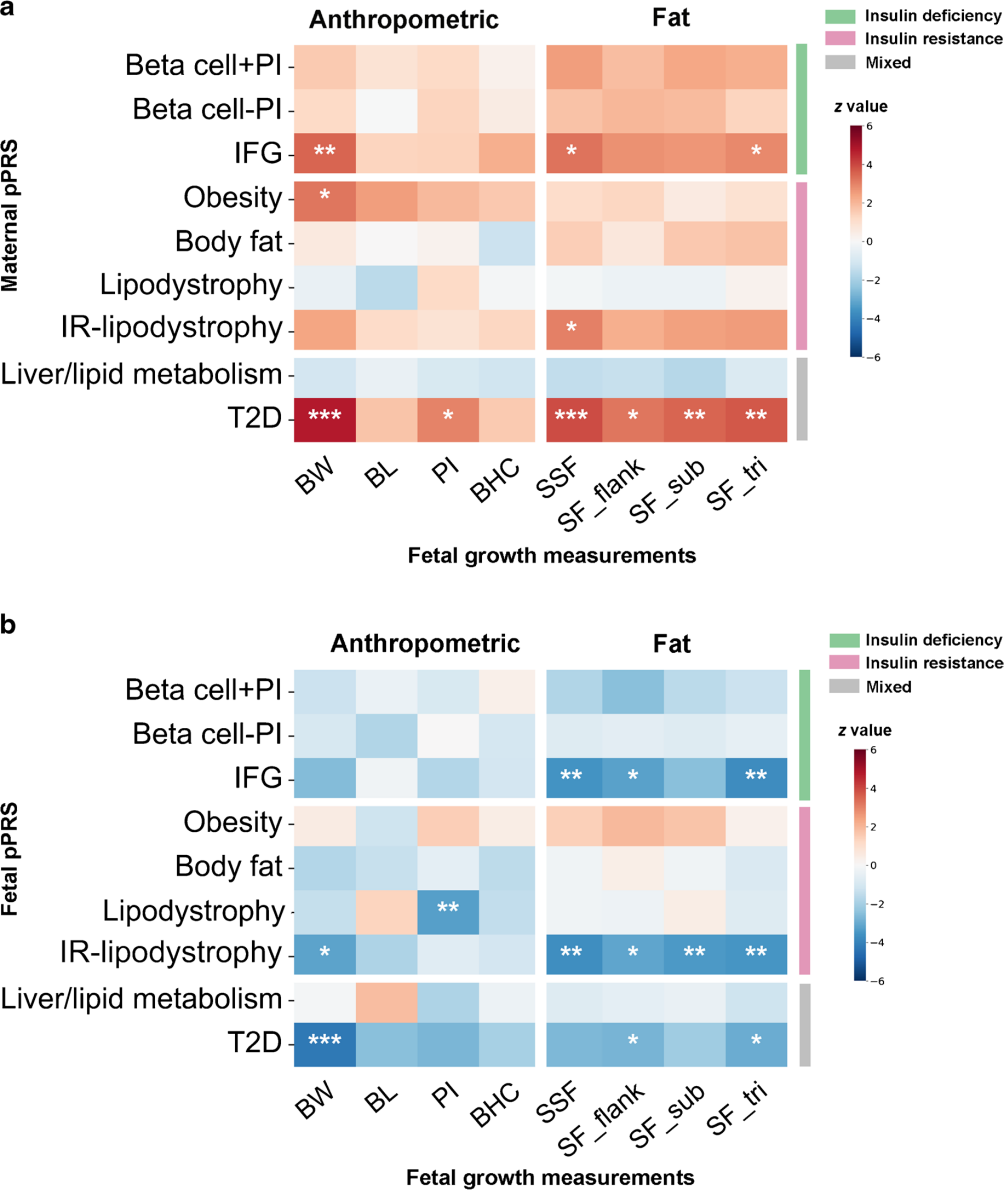
(**a**) Conditional associations of maternal pPRSs with offspring growth measurements in the HAPO Study (meta-analysis). (**b**) Conditional associations of fetal pPRSs with offspring growth measurements in the HAPO Study (meta-analysis). The colour bar indicates the *z* value of the regression coefficient. Analyses adjusted for gestational age at delivery, parity, maternal age at delivery, newborn sex, fetal top-five PCs and field centre (only for European individuals). *p<0.05, **p<0.01, ***p<0.001 (Bonferroni corrected). The ‘residual glycaemic’ cluster was renamed ‘IFG’; the ‘metabolic syndrome’ cluster was renamed ‘IR-lipodystrophy’. BHC, birth head circumference; BL, birth length; BW, birthweight; SF_flank, flank skinfold thickness; SF_sub, subscapular skinfold thickness; SF_tri, triceps skinfold thickness; T2D, type 2 diabetes

The correlations between maternal and fetal type 2 diabetes PRS/pPRS were around 0.5 (ESM Table 7). Meta-analyses of HAPO data found that maternal and fetal type 2 diabetes PRSs had opposite effects on fetal growth (Fig. 1 and ESM Table 8). Maternal type 2 diabetes PRS was associated with higher birthweight (1 g of birthweight per SD PRS: β 31.1 [95% CI 18.4, 43.8], *p*=1.8 × 10^−6^) and SSF (1 cm of SSF per SD PRS: β 0.21 [95% CI 0.11, 0.32], *p*=8.4 × 10^−5^), while fetal type 2 diabetes PRS was associated with lower birthweight (1 g of birthweight per SD PRS: β −27.9 [95% CI −40.6, −15.1], *p*=1.9 × 10^−5^) and SSF (1 cm of SSF per SD PRS: β −0.15 [95% CI −0.26, −0.04], *p*=0.007).

For specific pathways, maternal and fetal genetic effects acted through common and different insulin deficiency and insulin resistance pathways. Maternal IFG (formerly termed ‘residual glycaemic’) pPRS was associated with higher offspring birthweight (β 22.5 [95% CI 9.9, 35.2], *p*=4.9 × 10^−4^), while fetal IFG pPRS was associated with lower SSF (β −0.13 [95% CI −0.21, −0.06], *p*=3.2 × 10^−4^). Obesity pPRS showed a maternal-specific effect, which was associated with higher offspring birthweight (β 21.0 [95% CI 8.1, 33.8], *p*=0.001). Fetal IR-lipodystrophy (metabolic syndrome) pPRS was associated with lower birthweight (β−27.3 [95% CI −43.9, −10.6], *p*=0.001) and SSF (β −0.14 [95% CI −0.21, −0.06], *p*=2.1 × 10^−4^). Fetal Lipodystrophy pPRS was associated with lower PI. The pPRSs from soft-clustering also showed similar results (ESM Table 9). Findings from the unadjusted approach were generally consistent with those from the adjusted approach, although the magnitudes of associations of type 2 diabetes PRS and specific pPRSs were stronger after adjusting maternal and fetal PRSs simultaneously (ESM Table 10, ESM Fig. 3).

For abnormal fetal growth outcomes (ESM Table 11, ESM Fig. 4), maternal type 2 diabetes PRS (OR 1.23 [95% CI 1.11, 1.37], *p*=1.3 × 10^−4^), IFG pPRS (OR 1.17 [95% CI 1.05, 1.30], *p*=0.004) and Obesity pPRS (OR 1.19 [95% CI 1.07, 1.32], *p*=0.002) were associated with a higher risk of an LGA birth.

Thompson and colleagues [54] previously found that fetal alleles (14 SNPs) predisposing to metabolically favourable adiposity were associated with birthweight. To investigate whether Thompson et al’s SNPs overlap with or are in high LD (*R*^2^>0.8) with Suzuki’s SNPs in each genetic cluster, we used the LDpair in LDlink [19] to examine the pair-wise LD scores between Thompson et al’s SNPs and Suzuki’s SNPs in each genetic cluster from the 1000 Genome Project European reference panel. We mapped the 14 SNPs to our genetic clusters and found that most of them overlapped, or were in high LD, with the SNPs in the lipodystrophy-related pathways (ESM Table 12).

### Type 2 diabetes PRS and pPRSs were associated with maternal post-load hyperglycaemia in pregnancy

**Fig. 2:**
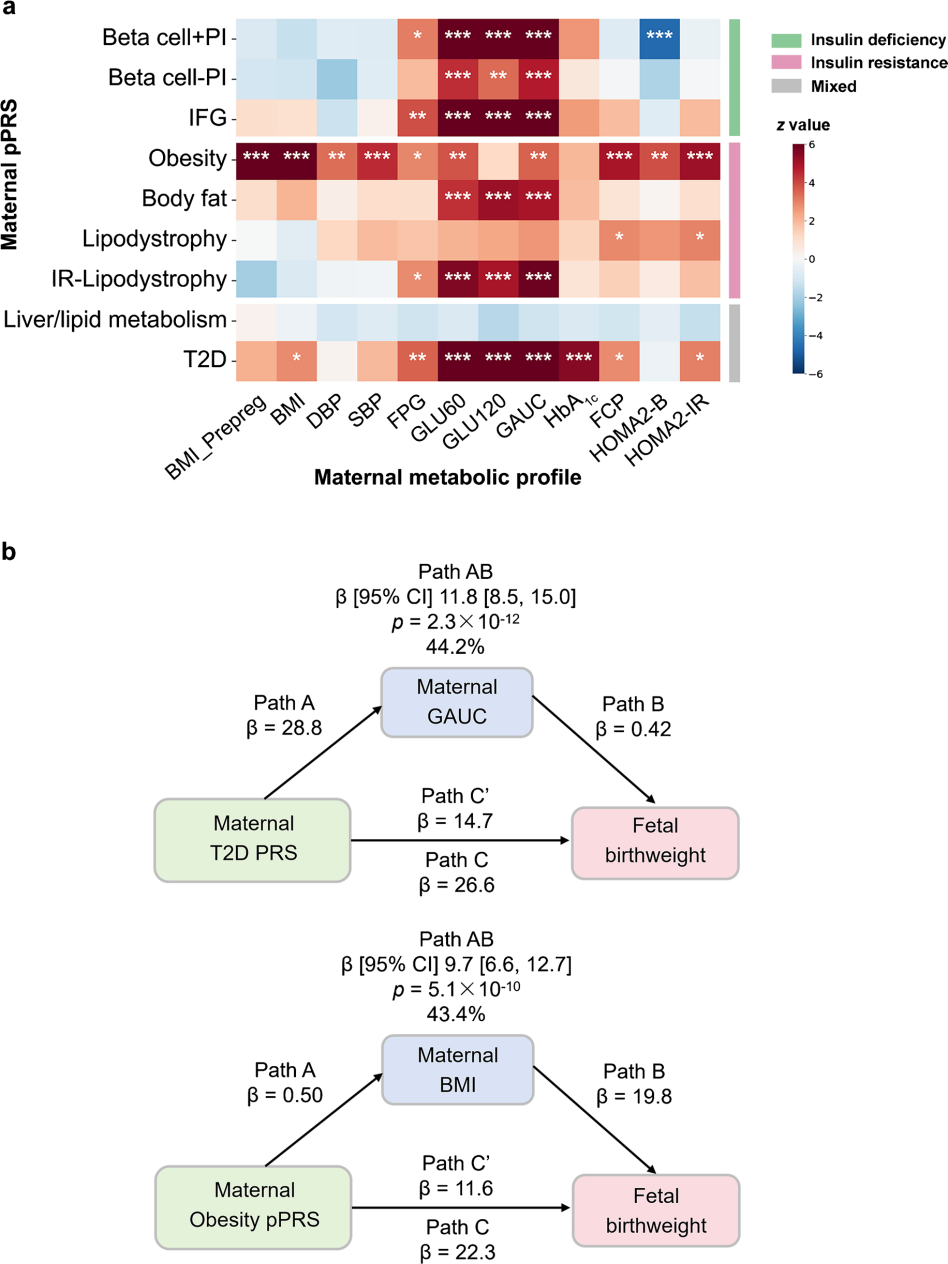
(**a**) Associations of maternal pPRSs with maternal metabolic profile in the HAPO Study (meta-analysis). The colour bar indicates the *z* value of the regression coefficient. Gestational age at OGTT, parity, maternal age and maternal top-five PCs and field centre (only for European individuals) were adjusted. *p<0.05, **p<0.01, ***p<0.001 (Bonferroni corrected). The ‘residual glycaemic’ cluster was renamed ‘IFG’; the ‘metabolic syndrome’ cluster was renamed ‘IR–lipodystrophy’. (**b**) Mediation (indirect) effect of maternal glucose levels (represented by GAUC) on the relationship between maternal type 2 diabetes PRS and offspring birthweight, and of maternal BMI on the relationship between maternal Obesity pPRS and offspring birthweight in HAPO (meta-analysis). Path AB represents the indirect effect. Path C represents the total effect without mediator. Path Cʹ represents the direct effect accounting for the indirect effect of mediator. The same covariates were adjusted. DBP, diastolic blood pressure; Prepreg, pre-pregnancy; SBP, systolic blood pressure; T2D, type 2 diabetes

The maternal type 2 diabetes PRS and pPRSs for Beta cell + PI, Beta cell − PI, IFG, Obesity, Body fat and IR-lipodystrophy all showed stronger associations with glucose levels at 1 h and 2 h and GAUC during the OGTT than with FPG (Fig. 2a, ESM Table 13) in the HAPO Study. The maternal Obesity pPRS was strongly associated with higher BMI before pregnancy and at third trimester, and with BP, FCP and HOMA2-IR.

### Ethnic differences in maternal and fetal genetic effects

Most of above associations showed low heterogeneity (*I*^2^<25% and Cochran’s *Q p*>0.05) across the different ethnic groups (ESM Tables 8–11, 13–16, ESM Figs. 6, 7). For maternal genetic effects on fetal growth outcomes, the most significant heterogeneity was seen between IFG pPRS and PI (*Q*=13.80*, p*_*Q*_=0.008*, I*^*2*^=0.71 [95% CI 0.26, 0.89]). Maternal IFG pPRS was associated with higher PI in Chinese and Mexican American groups but with lower PI in the African Caribbean group (ESM Table 8, ESM Fig. 8a). For fetal genetic effects, fetal IR-lipodystrophy pPRS was associated with lower birth length in Mexican American, African Caribbean and Thai groups but with higher birth length in the Chinese group (*Q*=13.45*, p*_*Q*_=0.009*, I*^*2*^=0.70 [95% CI 0.24, 0.88], ESM Table 8, ESM Fig. 8b). For the links between maternal PRSs and metabolic health in pregnancy, maternal type 2 diabetes PRS showed a much stronger association with higher FPG in European, African Caribbean and Chinese groups than in Mexican American and Thai groups (*Q*=16.99,*p*_*Q*_=0.002*, I*^*2*^=0.76 [95% CI 0.43, 0.90], ESM Fig. 8c).

### Maternal post-load hyperglycaemia and BMI mediated the maternal genetic effects

Mediation analysis showed that maternal post-load hyperglycaemia mediated the effect of maternal IFG pPRS on fetal birthweight. Maternal GAUC, reflecting maternal post-load hyperglycaemia, mediated 44.2% of the effect of the maternal type 2 diabetes PRS (1 g of birthweight per SD PRS through indirect effect: β 11.8 [95% CI 8.5, 15.0], *p*=2.3 × 10^−12^) and 34.2% of the effect of maternal IFG pPRS (β 8.0 [95% CI 5.5, 10.5], *p*=6.2 × 10^−10^) on offspring birthweight (Fig. 2 and ESM Fig. 9, ESM Table 14). Maternal BMI in pregnancy and before pregnancy mediated 43.4% (β 9.7 [95% CI 6.6, 12.7], *p*=5.1 × 10^−10^) and 26.3% (β 6.39 [95% 3.67, 9.12], *p*=4.3 × 10^−6^), respectively, of the effect of maternal Obesity pPRS on offspring birthweight. Maternal GAUC only mediated 7.9% (β 1.93 [95% CI 0.04, 3.82], *p*=0.045) of the effect of maternal Obesity pPRS on offspring birthweight (ESM Fig. 9). We did not find interaction effects between exposure and mediator on offspring birthweight (ESM Table 15).

Only the fetal Lipodystrophy pPRS was nominally associated with higher cord blood C-peptide (1 nmol/l of C-peptide per SD increase in pPRS: β 0.007 [95% CI 0.001, 0.013], *p*=0.019) (ESM Table 16, ESM Fig. 5) and did not survive the Bonferroni correction. Therefore, we did not perform the mediation analysis for cord blood C-peptide.

### 2SMR found consistent causal effects

**Fig. 3:**
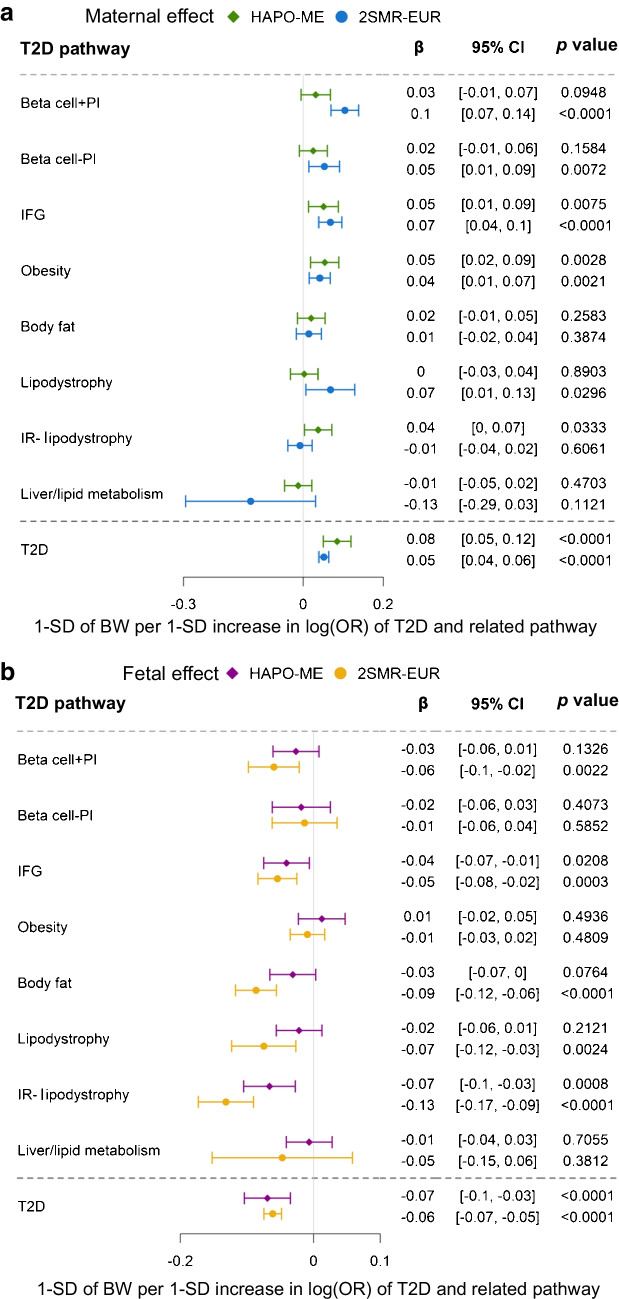
The maternal (**a**) and fetal (**b**) causal effects of type 2 diabetes-related pathways on offspring birthweight in HAPO-ME data and 2SMR-EUR. The birthweight in each HAPO ethnic group was *z* score transformed (per SD) in line with the European birthweight GWAS from EGG Consortium and UKB. The ‘residual glycaemic’ cluster was renamed ‘IFG’; the ‘metabolic syndrome’ cluster was renamed ‘IR-lipodystrophy’. BW, birthweight; T2D, type 2 diabetes

The effect size for maternal and fetal PRSs in 2SMR in European participants (2SMR-EUR) and multi-ethnic HAPO (HAPO-ME) data showed consistent directions and similar magnitudes (Fig. 3 and ESM Table 17). Maternal genetic predispositions to IFG and Beta cell + PI were associated with higher offspring birthweight. One SD increase in IFG-related type 2 diabetes risk log_*e*_ (OR) was causally associated with higher offspring birthweight (β 0.07 [95% CI 0.04, 0.10], *p*=4.8 × 10^−6^). Fetal genetic predisposition to lipodystrophy-related insulin resistance (IR-lipodystrophy) showed the strongest association with lower birthweight (β −0.13 [95% CI −0.17, −0.09], *p*=5.4 × 10^−10^). We also observed differences in effect size between the two approaches. The associations of type 2 diabetes PRS in 2SMR-EUR had smaller CIs than those in HAPO-ME. For the Beta cell + PI pathway, the associations with offspring birthweight were stronger in 2SMR-EUR than in HAPO-ME study. For the Body fat, Lipodystrophy and IR-lipodystrophy pathways, fetal effects on birthweight were also stronger in 2SMR-EUR than in the HAPO-ME study.

### Fetal corrected insulin response and ISI were causally associated with higher birthweight

**Fig. 4:**
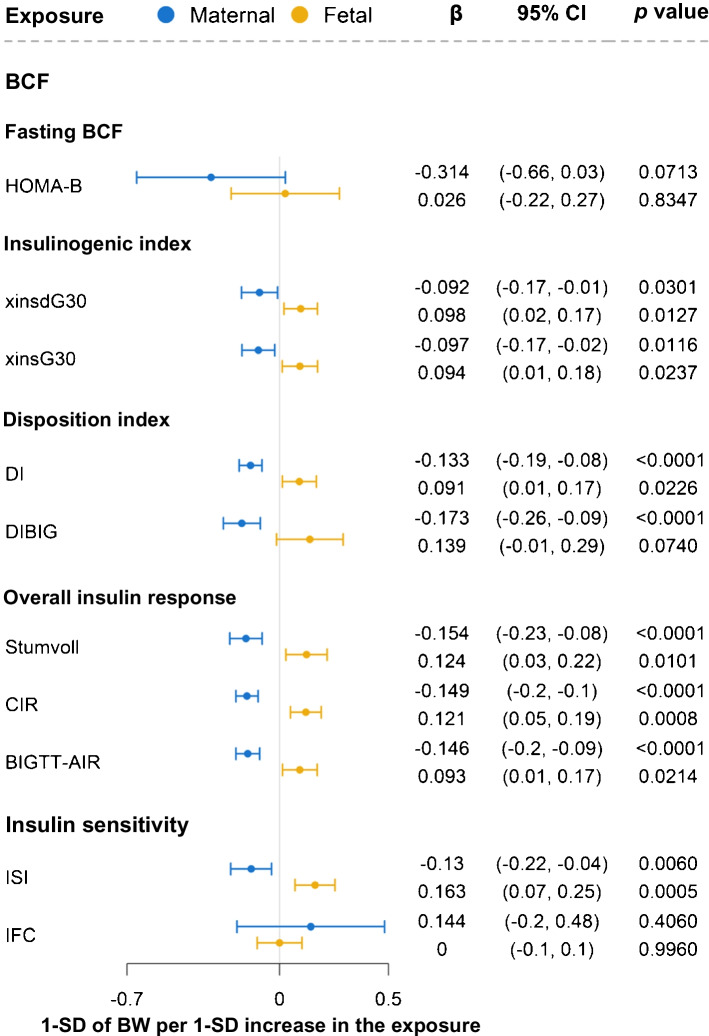
Effects of maternal and fetal BCF and insulin sensitivity indices on offspring birthweight by 2SMR. The exposures (except BIGTT-AIR and DIBIG) were adjusted for BMI. BIGTT-AIR, BW, birthweight; DI, disposition index for modified insulinogenic index and Matsuda; Stumvoll, first-phase Stumvoll

2SMR found that maternal overall insulin response (BIGTT-AIR, CIR and Stumvoll) and disposition index (DI and DIBIG) were causally associated with lower offspring birthweight (Fig. 4 and ESM Table 18). Fetal CIR (overall insulin response) (1 SD of birthweight per SD increase in genetically determined CIR: β 0.12 [95% CI 0.05, 0.19], *p*=7.8 × 10^−4^) and insulin sensitivity (ISI) (1 SD of birthweight per SD increase in genetically determined ISI: β 0.16 [95% CI 0.07, 0.25], *p*=4.7 × 10^−4^) was causally associated with higher birthweight.

### Shared causal variants act through distinct pathways

Co-localisation analyses identified 42 shared causal variants in 25 loci with fetal genetic effects and 53 shared causal variants in 13 loci with maternal genetic effects (Table 2 and ESM Table 19). Only *CDKAL1* showed both maternal and fetal genetic effects on birthweight. For specific pathways, the IFG (*n*=11) and IR-lipodystrophy (*n*=6) pathways had the most shared causal variants with fetal determined birthweight, while Beta cell + PI (*n*=25) and IFG (*n*=13) had the most shared causal variants with maternal determined birthweight. We found that most loci seemed to act through one pathway. Only a few loci acted through multiple pathways. In particular, *CDKAL1*, *TCF7L2*, *ADCY5* and *MACF1* acted through both insulin deficiency and insulin resistance pathways.

**Table 2:** Shared causal variants (PPH_4_ >0.75) between exposures (type 2 diabetes pathways, CIR and ISI) and maternal and fetal genetically determined birthweight by co-localisation analyses

Exposure	ID- or IR-related pathway	Maternal or fetal genetic effects		
		Maternal	Fetal	Common
Beta cell + PI	ID	*CDKAL1*, *TCF7L2*, *MTNR1B*, *CAMK2B*, *RMST*,* AEBP1*	*CDKAL1*,* ADCY5*	*CDKAL1*
Beta cell − PI	ID	*MTNR1B*,* CAMK2B*	*NKX6–3*, *ANK1*,* PIM3*	
IFG	ID	*CDKAL1*, *TCF7L2*, *MTNR1B*, *SRGAP2*, *RREB1*,* SSR1*	*CDKAL1*, *NKX6–3*, *MACF1*, *SFRP1*, *GPAT4-AS1*, *NALT1*, *MEG3*, *PANX2*,* NYAP2*	*CDKAL1*
Obesity	IR		*MACF1*, *DENND1A*,* MIR412*	
Body fat	IR	*CDKAL1, TCF7L2, TCF12, URGCP-MRPS24*	*CDKAL1*, *ADCY5*,* LCORL*	*CDKAL1*
Lipodystrophy	IR	*CYCSP55, HMGA1*	*IRS1*, *LINC01214*,* PEPD*	
IR-lipodystrophy	IR	*CDKAL1, TCF7L2, TCF12*	*CDKAL1*, *GPSM1*, *PLEKHA1*, *SOS2*, *LINC00880*,* RABGAP1*	*CDKAL1*
CIR	ID		*CDKAL1*, *GPSM1*, *HHEX*,* PLUT*	
ISI	IR	*COBLL1*	*IRS1*	

ID, insulin deficiency; IR, insulin resistance

## Discussion

Integrating a multi-ethnic cohort study with 2SMR analyses in Europeans, our study revealed differential pathways wherein maternal and fetal genetic predispositions to insulin deficiency and insulin resistance affect fetal growth. Maternal genetic predispositions to IFG-related insulin deficiency (mainly mediated by post-load hyperglycaemia) and obesity-related insulin resistance (mainly mediated by BMI) resulted in increased fetal growth. Fetal genetic predispositions to lipodystrophy-related insulin resistance and IFG-related insulin deficiency led to reduced fetal growth. Co-localisation analysis identified that some loci with shared causal variants, including *CDKAL1*, *TCF7L2*, *ADCY5* and *MACF1*, acted through both insulin deficiency and insulin resistance pathways.

Our findings support and further expand the genetic basis of the fetal insulin hypothesis [8–10] into multi-ethnic populations. Previous findings suggest that the relationships between insulin resistance/deficiency genes and birthweight are heterogeneous [8, 55]. We found evidence supporting the notion that fetal insulin resistance secondary to lipodystrophy is a key genetic pathway impacting reduced birthweight and fat deposition. Furthermore, using insulin response and sensitivity measurements derived in non-diabetic individuals, we found that fetal insulin sensitivity was causally associated with higher birthweight. Although the Lipodystrophy pathway is more strongly associated with reduced body and trunk fat than the IR-lipodystrophy pathway, the fetal Lipodystrophy pathway only showed an association with reduced PI, and not fetal fat, in the HAPO Study and showed a modest association with birthweight in 2SMR. This indicates that insulin resistance secondary to lipodystrophy, rather than lipodystrophy alone, may have a greater impact on fetal growth and fat deposition. In addition, these observed associations can be attributed to shared causal variants in *CDKAL1*, *GPSM1*, *PLEKHA1* and *HHEX*. Although these loci have been previously reported to be associated with lower birthweight [8], our findings suggest that they may act through insulin resistance secondary to lipodystrophy.

The maternal and fetal genetic effects on birthweight and SSF were generally consistent across five ethnic groups in our study. Nevertheless, we observed some ethnicity differences in pathway-specific genetic effects on other fetal growth measurements, including PI and birth length, which might have an ethnicity-specific genetic basis or be attributed to other environmental factors affecting specific growth outcomes. For example, although most participants in Chinese and Thai groups belong to East Asian ancestry, the direction of the association between fetal IR-lipodystrophy pPRS and birth length in the Chinese group was opposite to that in the Thai group.

We found that the type 2 diabetes PRS and IFG pPRS were more strongly associated with post-load hyperglycaemia than fasting hyperglycaemia in pregnancy, suggesting that dynamic insulin response and peripheral insulin sensitivity in pregnancy were more affected by genetic variants associated with type 2 diabetes and IFG compared with basal hepatic glucose regulation. In addition, although the IFG genetic cluster is only modestly associated with elevated FPG in the general populations [15], it displayed much stronger effects on post-load hyperglycaemia in pregnancy, indicating the shared genetic basis between IFG-related insulin deficiency in the general population and post-load hyperglycaemia in pregnancy. Furthermore, maternal type 2 diabetes PRS, IFG pPRS and Obesity pPRS were also associated with a higher risk of LGA birth. Post-load hyperglycaemia and BMI in pregnancy mainly mediated the maternal genetic effects of type 2 diabetes/IFG and obesity, respectively. This suggests a potential precision intervention strategy for LGA offspring: target pregnant women with higher genetic predispositions to type 2 diabetes and specific pathways, and aim to reduce their post-load hyperglycaemia and BMI in pregnancy.

Co-localisation analyses identified type 2 diabetes risk loci differentially sharing causality with fetal and maternal genetically determined birthweight and further revealed their underlying biological pathways. We confirmed the causal effects of some previously reported type 2 diabetes loci on birthweight, including *HHEX* [13, 56, 57], *CDKAL1* [13, 57–61], *ADCY5* [13, 59–61], *ANK1* [8, 62, 63], *IRS1* [8, 62, 63], *MTNR1B* and *TCF7L2* [2, 35, 57], and highlighted *CDKAL1* as the only locus with both maternal and fetal effects. Besides, our results identified that some loci may act through both insulin deficiency and insulin resistance pathways, including *CDKAL1*, *TCF7L2*, *ADCY5* and *MACF1*, suggesting the complex biological mechanisms behind the identified causality.

In this study, we integrated pPRS with the emerging cluster-stratified MR approach [33, 64, 65] to dissect the heterogeneity of type 2 diabetes as well insulin deficiency and insulin resistance, and to reveal the biological pathways driving the previously identified relationships. Mediation analysis further quantified the extent to which the intrauterine metabolic environment contributes to maternal genetic effects on fetal growth. In parallel to the recent advances in precision diabetes medicine [14, 66–68], our findings shed light on the clinical manifestations of distinct genetic subtypes of type 2 diabetes in the very early stage of life, and may hence help understand the developmental origins of diabetes heterogeneity.

We acknowledge that there are some limitations in our study. We observed little evidence of associations between type 2 diabetes PRS/pPRSs and cord blood C-peptide. Furthermore, previous PRS analyses did not find any association of PRSs for birthweight [28] and metabolically favourable adiposity [54] with cord blood C-peptide either. It is possible that the lack of associations reflects a true lack of effects of these PRSs on fetal insulin. However, this is unlikely, given the known associations of several of these PRSs with insulin indices in adults [16, 19, 20]. Instead, it is possible that the lack of associations is due to low statistical power of PRSs and/or the fact that cord blood C-peptide measured at birth is an imperfect proxy for fetal insulin secretion or resistance occurring during gestation. Larger sample size will be required to investigate this further.

The findings from the five ethnic groups in our study may not be applicable to other under-represented populations not included in our study, such as South Asian and Middle Eastern populations. Our MR analyses on birthweight were restricted to European populations due to the limited availability of GWAS data sources. Therefore, the MR results should be interpreted with caution. There has not been large GWAS summary data for SSF or other infant fat distribution measurements. Thus, we cannot validate our current findings using 2SMR on infant fat. In addition, we only used static indices for insulin deficiency and insulin resistance in pregnancy based on HOMA2. Dynamic indices such as ISI [69] are better to capture maternal dynamic insulin response and sensitivity.

The conditional analyses adjusting for maternal and fetal PRS could lead to a collider bias [70, 71]. Including paternal genotype as a negative control or examining the parent-of-origin effects through haplotype phasing on mother–child duos can address this issue, which can be our future work. Nevertheless, previous simulation studies [2, 70, 72, 73] have found that adjusting for offspring genetics only leads to a modest negative bias for maternal genetic effect. In contrast, failing to adjust for offspring genetics can lead to a large positive bias (spurious association) for maternal genetic effect. Our finding from an unadjusted approach suggests that if maternal or fetal PRS was to be examined on its own, the indirect intrauterine effect and direct fetal genetic effect can confound each other [4]. However, performing conditional analyses do help to disentangle the direct fetal genetic effect from the indirect intrauterine effect.

In conclusion, genetic predispositions to lipodystrophy-related insulin resistance and IFG-related insulin deficiency result in reduced fetal growth. Targeting pregnant women with high type 2 diabetes PRS/pPRS and prescribing interventions to reduce their post-load hyperglycaemia and BMI may help to reduce offspring risk of LGA birth.

## Supplementary Information

Below is the link to the electronic supplementary material.

ESM (PDF 2346 KB)

ESM tables (XLSX 571 KB)

## Data Availability

Genotype and clinical data from HAPO centres other than Hong Kong were obtained from the database of Genotypes and Phenotypes (dbGaP; https://www.ncbi.nlm.nih.gov/projects/gap/cgi-bin/study.cgi?study_id=phs000096.v4.p1) with accession number ‘phs000096.v4.p1’. The GWAS summary statistics can be downloaded from http://www.diagram-consortium.org/downloads.html (type 2 diabetes), https://egg-consortium.org/ (maternal and fetal genetically determined birthweight), https://kp4cd.org/node/1559 (insulin secretion) and https://magicinvestigators.org/downloads/ (insulin sensitivity).

## Abbreviations

2SMR: Two-sample Mendelian randomisation
2SMR-EUR: 2SMR in European participants
BCF: Beta cell function
BIGTT-AIR: BCF insulin sensitivity GTT for acute insulin response
BIGTT-SI: BCF insulin sensitivity GTT for ISI
CIR: Corrected insulin response
dbGaP: Database of Genotypes and Phenotypes
DI: Disposition index
DIBIG: Disposition index for BIGTT-AIR and BGTT-SI
FCP: Fasting C-peptide
FPG: Fasting plasma glucose
GAUC: AUC for glucose
GLU120: 2 h glucose
GLU60: 1 h glucose
GWAS: Genome-wide association study
HAPO: Hyperglycaemia and Adverse Pregnancy Outcome
HAPO-ME: Multi-ethnic HAPO
HCC: Head circumference
IFC: Insulin fold change
IFG: Impaired fasting glucose
IR-lipodystrophy: Insulin resistance–lipodystrophy
ISI: Modified Stumvoll insulin sensitivity index
IVW: Inverse variance-weighted
LD: Linkage disequilibrium
LGA: Large-for-gestational-age
MR: Mendelian randomisation
PC: Principal component
PI: Ponderal index
PPH_4_: Posterior probability of hypothesis 4
pPRS: Partitioned polygenic risk score
PRESSO: Pleiotropy Residual Sum and Outlier
PRS: Polygenic risk score
QC: Quality control
SGA: Small-for-gestational-age
SSF: Sum of skinfold thicknesses
xinsdG30: Insulinogenic index
xinsG30: Modified insulinogenic index

## Code availability

The codes for all the analysis in this study are stored in GitHub https://github.com/CharlesLambert70/Fetal-growth-analysis

The following software and packages were used for data analysis:

R packages: coloc: https://cran.r-project.org/web/packages/coloc/index.html; meta: https://cran.r-project.org/web/packages/meta/index.html; metapower: https://cran.r-project.org/web/packages/metapower/index.html; LDlinkR: https://cran.r-project.org/web/packages/LDlinkR/index.html; TwoSampleMR: https://mrcieu.github.io/TwoSampleMR/; MATLAB toolbox: Mediation: https://github.com/canlab/MediationToolbox
