# Supplementary material for: Maternal and fetal genetic predispositions to insulin deficiency and resistance affect fetal growth through distinct pathways
Source: Diabetologia. 2026 Feb 3;69(7):1935–50. doi: 10.1007/s00125-026-06669-7 (PMC13236813; doi:10.1007/s00125-026-06669-7)
Supplement: Supplementary file 1 — ESM (PDF 2346 KB) [file 125_2026_6669_MOESM1_ESM.pdf]

Supplemental materials

Supplemental methods

Genotyping, quality control and imputation

We applied uniform quality control (QC) procedures on the maternal and fetal genotype data of each ethnic group: (1) verification of sex based on genotype calls from chromosomes X and Y; (2) identification of low-quality samples based on call rate and heterozygosity rate; (3) detection of potential familial relationships or duplicated individuals using estimates of identity-by-descent; and (4) assessment of population stratification through principal component (PC) analysis. Only biallelic autosomal single nucleotide polymorphisms (SNPs) were included in the per-marker QC. SNPs were excluded from further analysis if they met any of the following criteria: (1) Hardy–Weinberg equilibrium $P < 1\times10^{-4}$; (2) minor allele frequency (MAF) <1%; or (3) call rate <95%. In particular, SNPs with MAF ≥1% but ≤5% were excluded if their call rate was <99%.

Genetic ancestry inference

We merged the maternal and fetal genotype data with Phase 3 of 1000 Genomes (1KG) Project reference samples. Then a random forest classifier with five ancestry categories, including European (EUR), East Asian (EAS), South Asian (SAS), African (AFR), and Admixed American (AMR), was trained on the merged genotype data for maternal and fetal genotypes in each HAPO ethnic group, respectively, using the top 20 genetic principal components (PCs). Genetic ancestry for each individual was determined by a predicted probability > 0.8. Please see the Supplemental Figure S1 and

S2 for the distribution plot of top two PCs for each individual. Most European participants were assigned to European ancestry; Most Mexican-American participants were assigned to Admixed American/Hispanic ancestry; Most Afro-Caribbean participants were assigned to African ancestry; Most Chinese and Thai participants were assigned to East Asian ancestry.

Figures

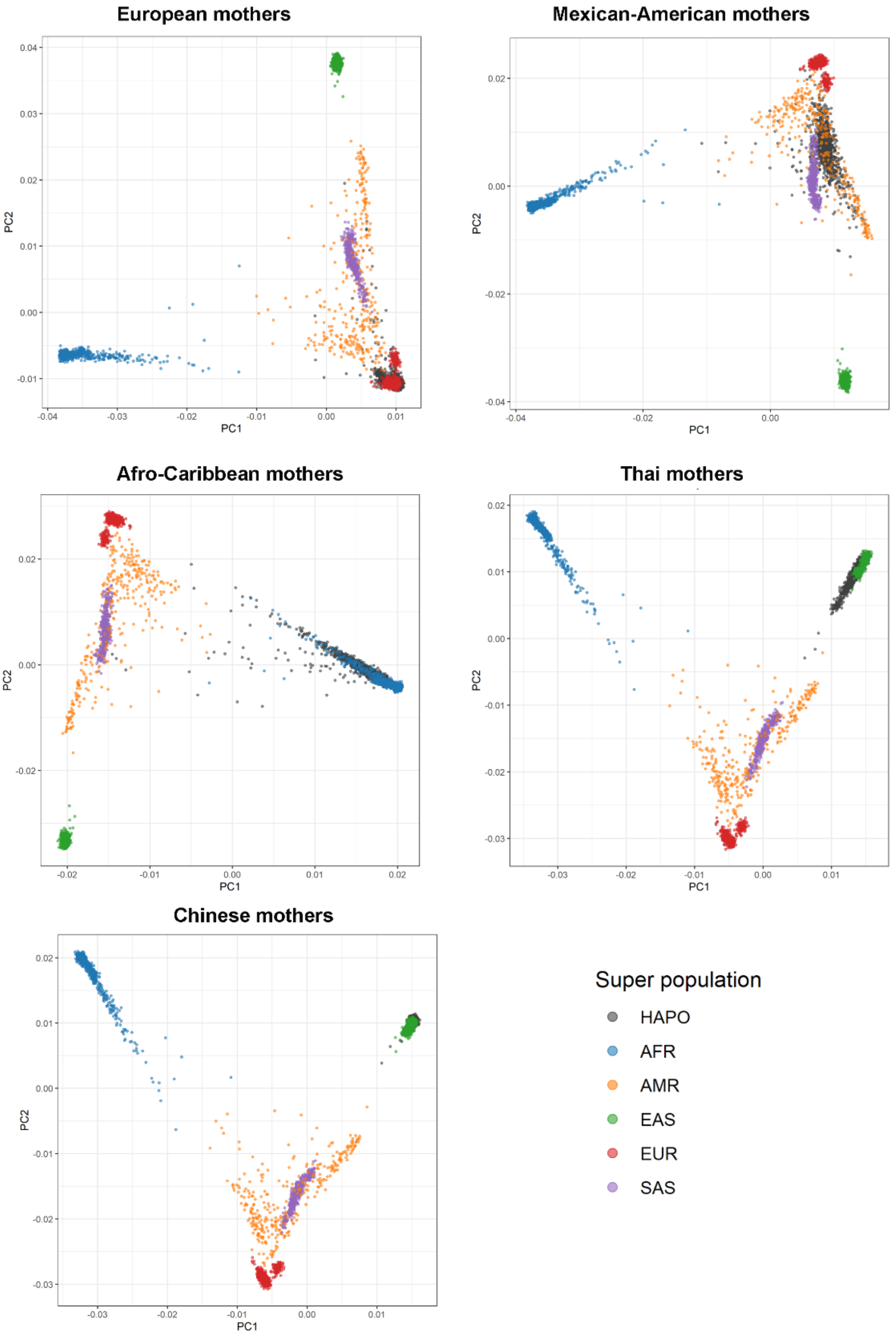

Figure 1. Distribution of top 2 PCs for each mother from the five ethnic groups. X axis is PC1, while Y axis is PC2. The blue (African ancestry), orange (Admixed American ancestry), green (East Asian ancestry), red (European ancestry) and purple (South Asian ancestry) dots are subjects from the five superpopulations from 1KG. Grey dots are mothers in each HAPO ethnic group.

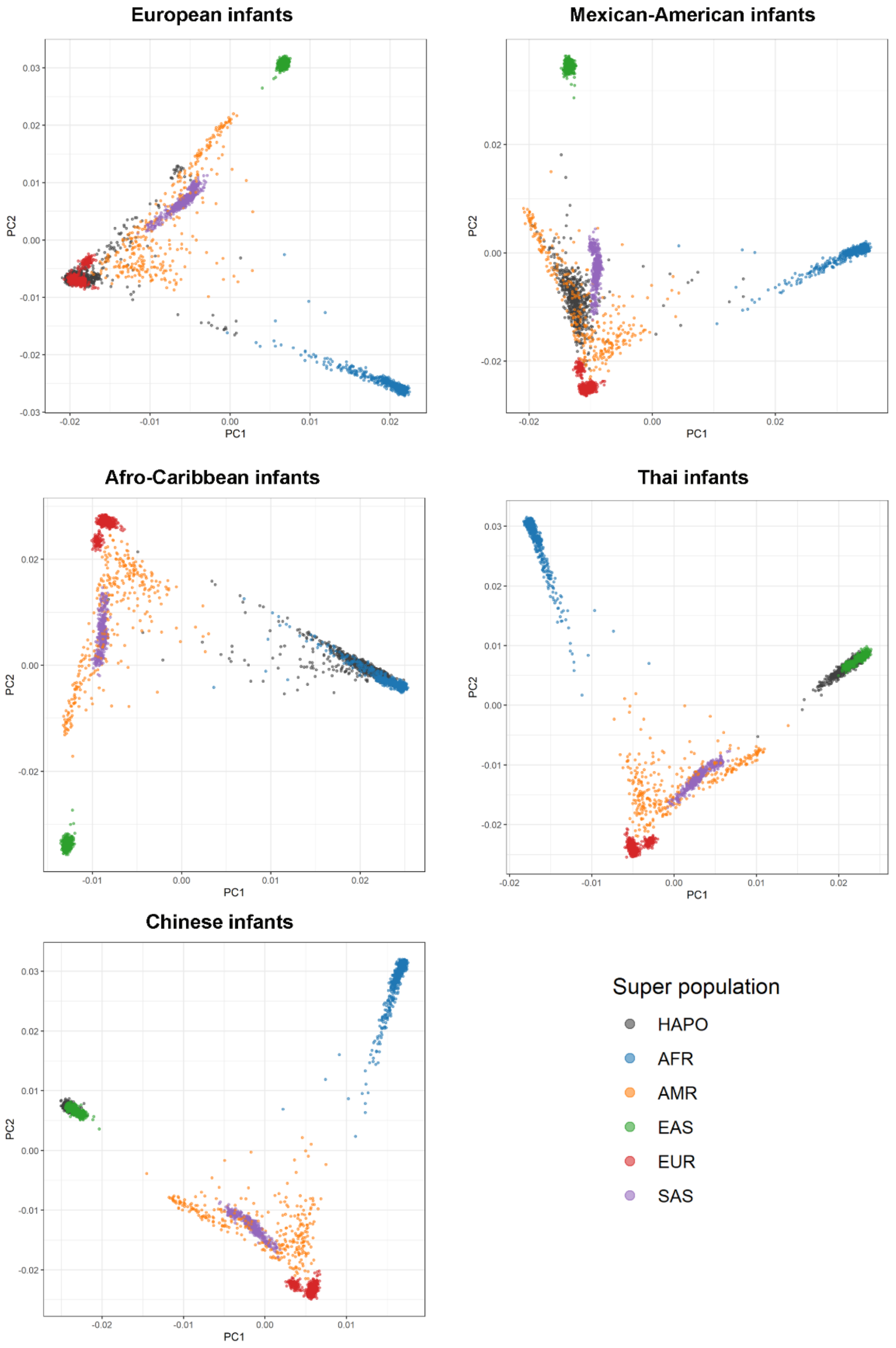

Figure 2. Distribution of top 2 PCs for each infant from the five ethnic groups. X axis is PC1, while Y axis is PC2. The blue (African ancestry), orange (Admixed American ancestry), green (East Asian ancestry), red (European ancestry) and purple (South Asian ancestry) dots are subjects from the five superpopulations from 1KG. Grey dots are infants in each HAPO ethnic group.

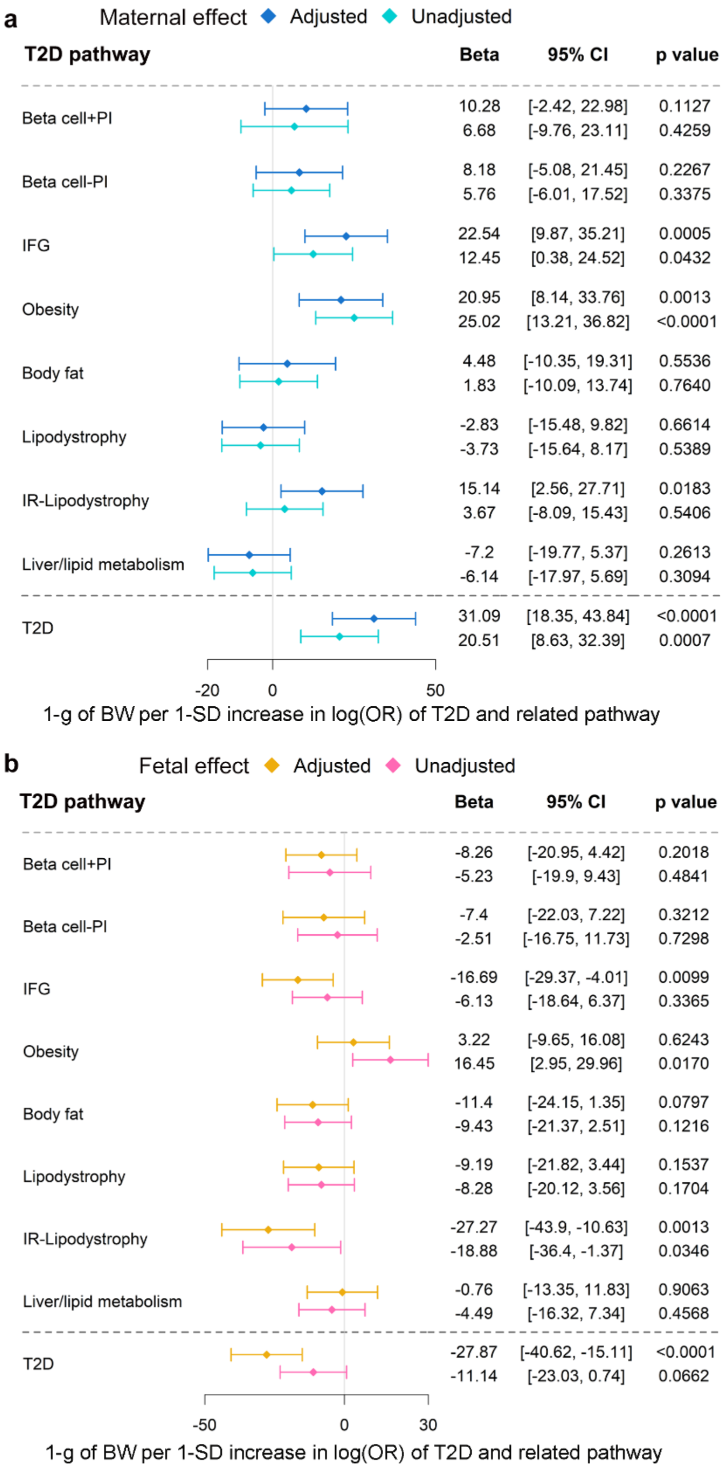

Figure 3. Associations of **(a)** maternal and **(b)** fetal T2D PRS and pPRSs with birth weight adjusted simultaneously or unadjusted for maternal and fetal PRS in HAPO multi-ancestry data.

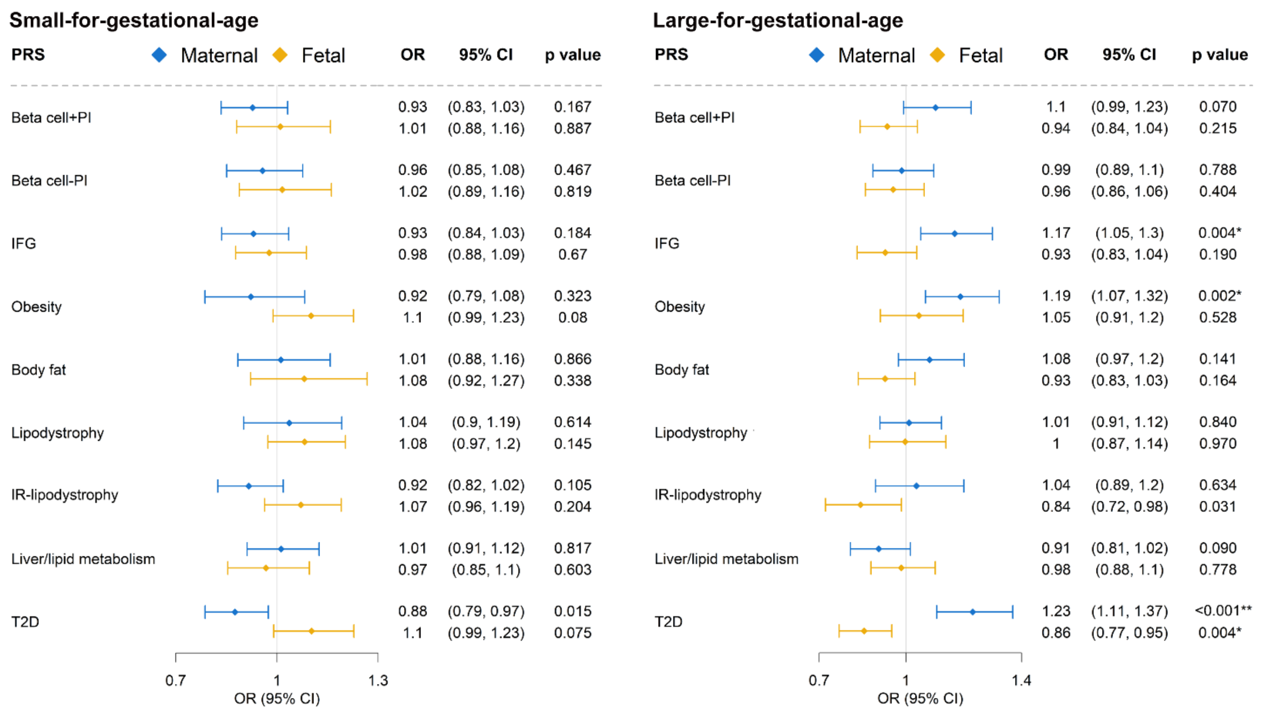

Figure 4. Conditional associations of maternal and fetal pPRSs with small-for-gestational-age and large-for-gestational-age in the HAPO Study (meta-analysis). Analyses adjusted for gestational age at delivery, parity, maternal age at delivery, newborn sex, fetal top five PCs and field center (only for European individuals). *, Bonferroni corrected p <0.05; **, Bonferroni corrected p <0.01; ***, Bonferroni corrected p <0.001.

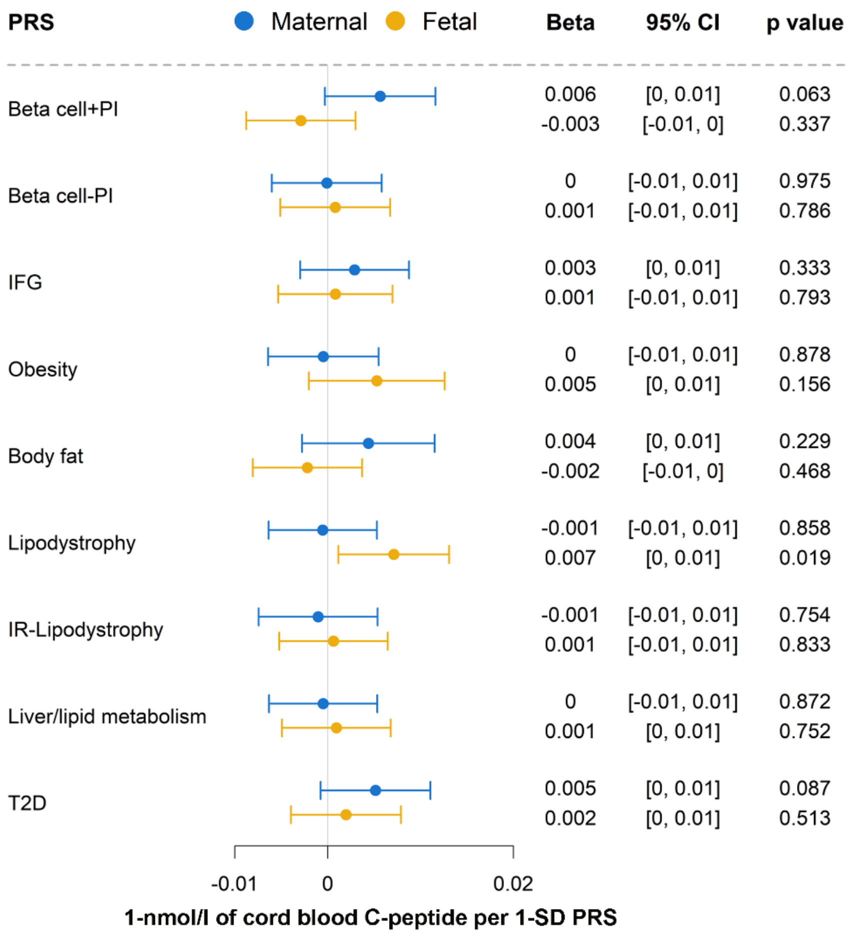

Figure 5. The associations of maternal and fetal pPRSs with cord blood C-peptide (nmol/l) in the HAPO Study (meta-analysis). Field center, gestational age at delivery, parity, maternal age at delivery, newborn sex and fetal top five PCs were adjusted. The error bars indicate 95% CI.

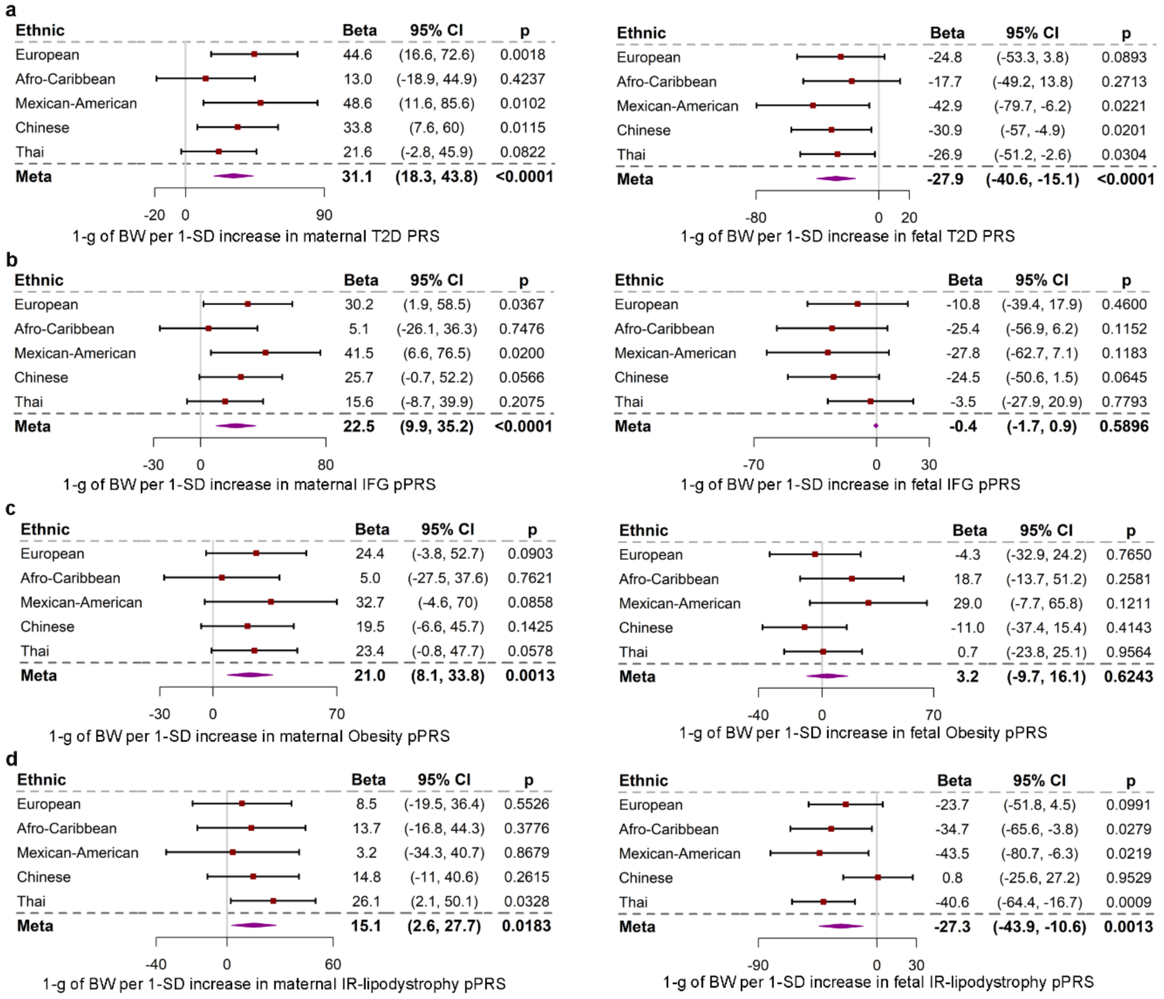

Figure 6. Associations of T2D PRS/pPRSs with outcomes in five ethnic groups. **(a)** Associations of maternal and fetal T2D PRS with BW. **(b)** Associations of maternal and fetal IFG (impair fasting glucose) pPRS with BW. **(c)** Associations of maternal and fetal Obesity pPRS with BW. **(d)** Associations of maternal and fetal IR-lipodystrophy pPRS with BW. Analyses adjusted for gestational age at delivery, parity, maternal age at delivery, newborn sex, fetal top five PCs and field center (only for European individuals).

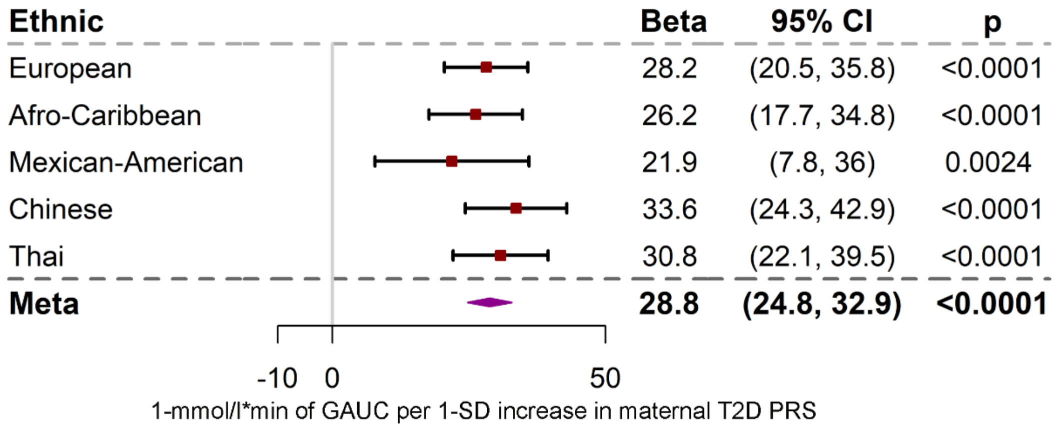

Figure 7. Associations of maternal and fetal T2D PRS with maternal GAUC during OGTT in five ethnic groups. Gestational age at OGTT, parity, maternal age and maternal top five PCs and field center (only for European individuals) were adjusted.

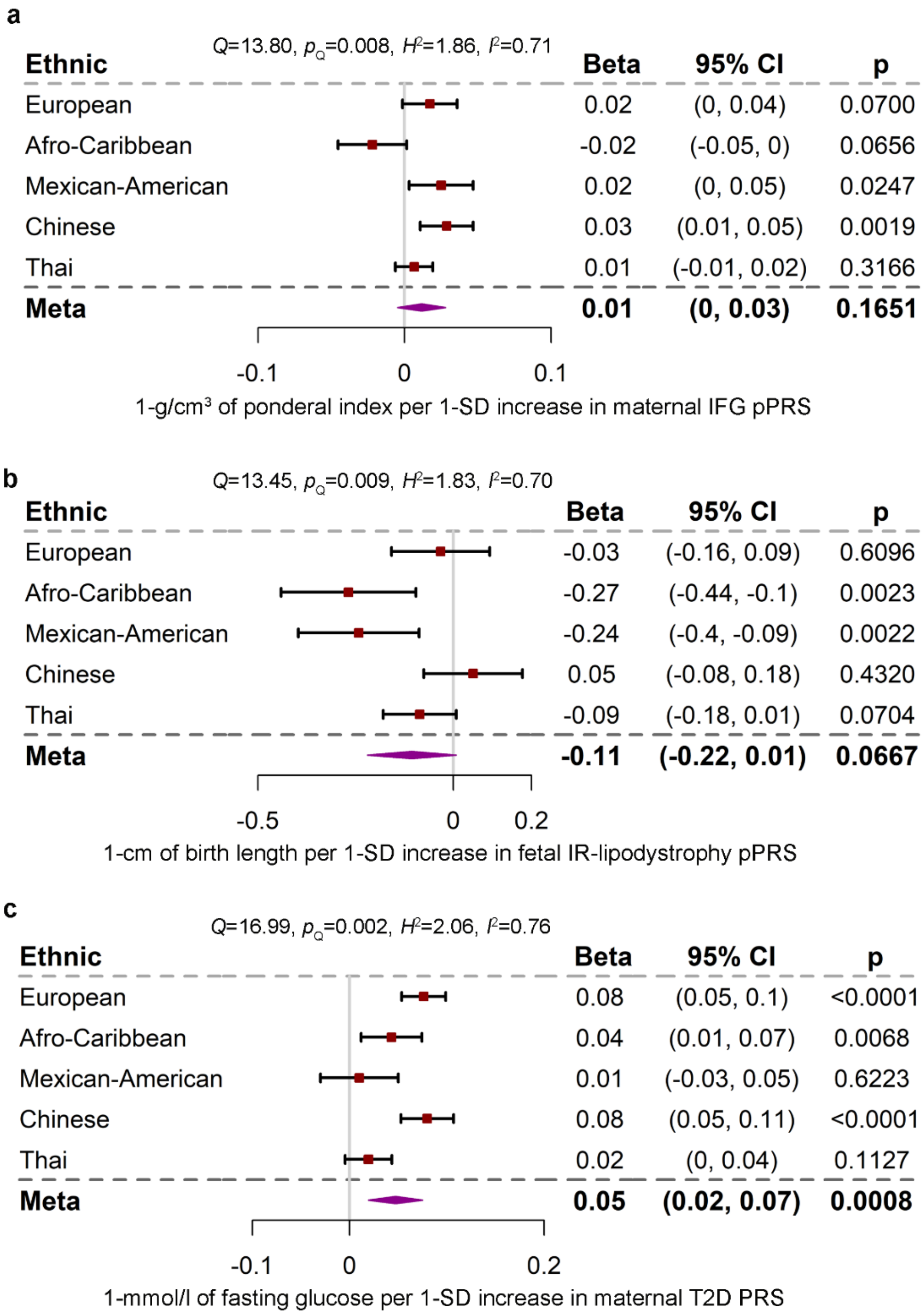

Figure 8. Associations of T2D PRS/pPRSs with outcomes in five ethnic groups. **(a)** Associations of maternal IFG pPRS with ponderal index. **(b)** Associations of fetal IR-lipodystrophy pPRS with birth length. **(c)** Associations of maternal T2D PRS with fasting glucose.

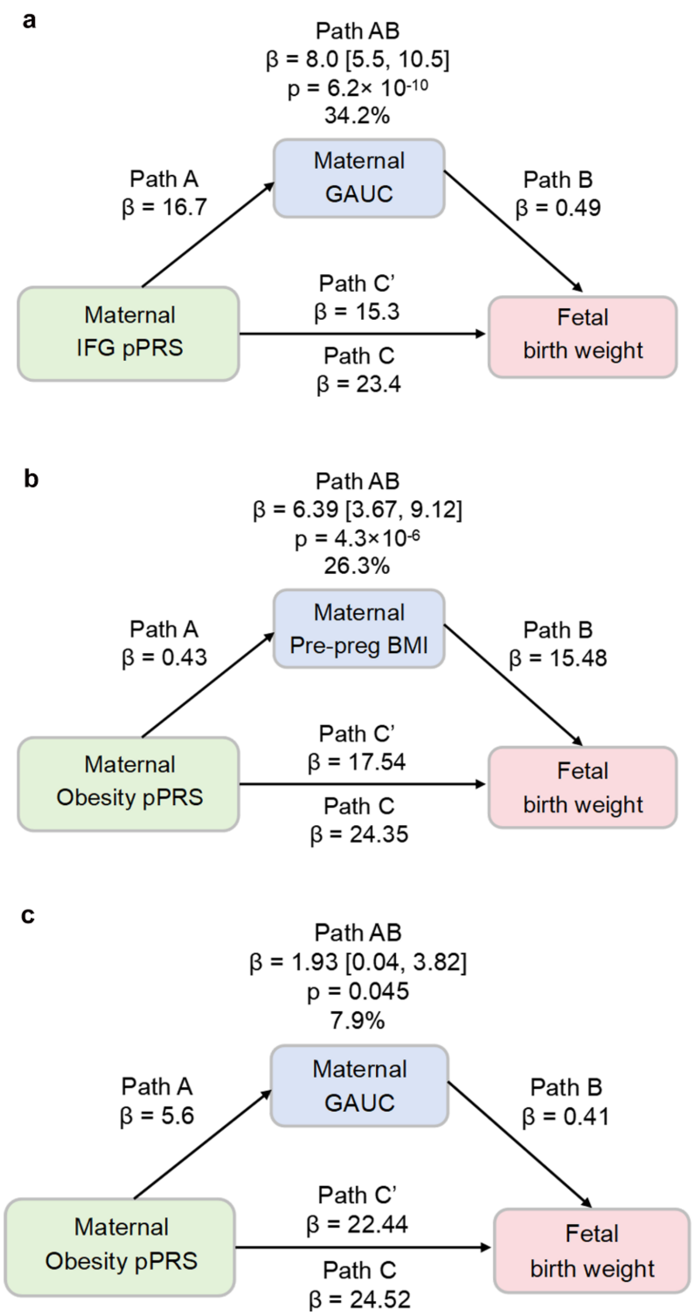

Figure 9. (a) Mediation effect of maternal GAUC on the association between IFG pPRS and fetal birth weight. **(b)** Mediation effect of maternal BMI before pregnancy on the association between obesity pPRS and fetal birth weight. Path AB represents the indirect effect. **(c)** Mediation effect of maternal GAUC on the association between Obesity pPRS and fetal birth weight. Path AB represents the indirect effect. Path C represents the total effect without mediator. Path C' represents the direct effect accounting for the indirect effect of mediator. Same covariates were adjusted.
